# Solution NMR Spectroscopy in Target-Based Drug Discovery

**DOI:** 10.3390/molecules22091399

**Published:** 2017-08-23

**Authors:** Yan Li, Congbao Kang

**Affiliations:** Experimental Therapeutics Centre, Agency for Science, Technology and Research (A*STAR), 31 Biopolis Way, Nanos, #03-01, Singapore 138669, Singapore; yli@etc.a-star.edu.sg

**Keywords:** NMR, drug discovery, hit identification, protein dynamics, protein-ligand interactions, fragment screening

## Abstract

Solution NMR spectroscopy is a powerful tool to study protein structures and dynamics under physiological conditions. This technique is particularly useful in target-based drug discovery projects as it provides protein-ligand binding information in solution. Accumulated studies have shown that NMR will play more and more important roles in multiple steps of the drug discovery process. In a fragment-based drug discovery process, ligand-observed and protein-observed NMR spectroscopy can be applied to screen fragments with low binding affinities. The screened fragments can be further optimized into drug-like molecules. In combination with other biophysical techniques, NMR will guide structure-based drug discovery. In this review, we describe the possible roles of NMR spectroscopy in drug discovery. We also illustrate the challenges encountered in the drug discovery process. We include several examples demonstrating the roles of NMR in target-based drug discoveries such as hit identification, ranking ligand binding affinities, and mapping the ligand binding site. We also speculate the possible roles of NMR in target engagement based on recent processes in in-cell NMR spectroscopy.

## 1. Introduction

Solution Nuclear Magnetic Resonance (NMR) spectroscopy has been used in analyzing structures of proteins, nucleic acids and small molecules [1,2,3]. In addition to determining protein structures, NMR spectroscopy is very useful for studying protein-ligand/protein interactions and protein dynamics [4,5,6]. This technique is especially powerful in elucidating biomolecules’ behavior under physiological conditions [7]. With the development of strategies for the preparation of isotopically labeled proteins, availability of high-field NMR magnets [8,9,10,11], and newly developed and optimized pulse programs [12,13,14], many protein-protein complexes with high molecular weight can be characterized using NMR spectroscopy [8,9,11]. In addition to the structural and dynamic characterization of a protein, NMR spectroscopy has been proven to be a very useful tool in the target-based drug discovery in the steps of hit identification and lead optimization [15,16,17]. It is a useful tool to validate the identified hits from high-throughput screening (HTS). Compared with other methods, multidimensional NMR using isotopically enriched proteins has a high potential for minimizing false positives in the study of protein-ligand interactions [18]. It can also be used to map the ligand/inhibitor binding site to facilitate structure-based drug design [19]. Fragment-based drug discovery (FBDD) is an alternative approach to HTS for finding hit compounds. It has been widely used in drug discovery projects [20,21,22,23]. NMR has been proven to be very powerful for screening fragments because of its capability to identify weak binding hits from the fragment libraries [20,24,25]. The identified hits can be further grown into more potent compounds based on biophysical and biochemical assays [26]. FBDD using NMR spectroscopy has been successful in some drug discovery projects and several drug candidates and potent inhibitors have been developed using this approach [27,28,29,30]. This approach has been described in several reviews [20,24,25,26,27,31,32,33,34,35,36,37,38] and will not be discussed here. Herein, we describe challenges of NMR technique in target-based drug discovery. We also show that NMR will play a critical role in the target-based drug discovery.

## 2. Challenges of NMR in Drug Discovery

**Figure 1:**
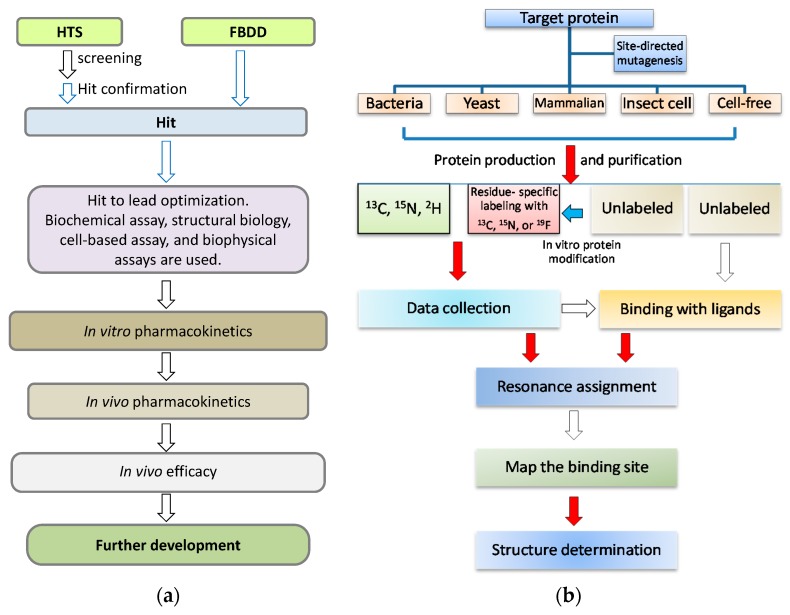
NMR in drug discovery. (**a**) A simplified flowchart in drug discovery process. When a target is defined, a couple of steps will be gone through in the drug discovery. NMR spectroscopy is useful and plays important roles in the early stage, which is highlighted with blue arrows; (**b**) A flowchart for the procedures in protein NMR studies. There are several challenging and time-consuming steps from target gene cloning to target-ligand complex structural determination, which include target protein purification, resonance assignment, and structure determination. The challenging steps are highlighted with red arrows.

In a target-based drug discovery process, NMR can play important roles in the early hit identification stage [37] (Figure 1a) while NMR might be useful for testing target-inhibitor interactions at the late stage of the drug development to confirm protein and lead interactions in the living cells [39,40]. Any NMR experiment (ligand-observed or protein-observed experiments) [41,42,43] that can probe protein and ligand interactions will be able to play the aforementioned roles (Table 1). It has been noted that suitable types of experiments can be chosen based on the ligand binding affinities, the experimental purposes, and sizes of the target proteins. Sample preparation is the key step in NMR studies before the experiments are carried out (Figure 1b). For most of the protein-observed heteronuclear experiments, isotopically labeled proteins are required. In addition, it would be ideal if the sample were stable under the experimental conditions. In summary, the challenges of NMR in drug discovery mainly include sample preparation, protein stability, and spectral quality of the target protein. Such challenges which are also applicable to membrane proteins whose folding in different systems will not be discussed in this review.

### 2.1. NMR Sample Preparation Challenges

NMR spectroscopy is a powerful tool to study protein structure and dynamics while challenges still remain for the targets with large molecular weight (>30 kDa) [3]. For a target with low production yield, poor stability or poor spectra quality under the experimental conditions, it will also be challenging to apply NMR to the project.

#### 2.1.1. Sample Preparation

Sample preparation is the key step for NMR studies and it is one of the challenging steps for NMR studies. The commonly used system for protein preparation is the *Escherichia coli* (*E. coli*) cells because of the low cost for cell growth and easy operational procedures [44,45]. Isotopic labeling of a target protein can be easily achieved with relatively low cost. Most NMR studies are using protein samples produced from *E. coli*. Some drug targets such as kinases, receptors, and ion channels are very difficult to be produced from *E. coli* cells due to their large size or requirement of post modifications such as phosphorylation and palmitoylation. Other protein expression systems include yeast [46], insect cells [47] and mammalian cells [48,49]. For some difficult targets such as membrane proteins, massive work has to be done to obtain a suitable condition for protein production [45,50]. Cell-free expression system is also shown to be efficient for protein production for NMR experiments [51,52,53]. Many proteins such as proteases and membrane-bound proteins are prepared for NMR studies using cell-free expression systems [54,55,56,57]. Cell-free expression of protein can also speed up backbone assignment by using a combinatorial labeling scheme [58]. Despite the expression system used for protein production, a fusion tag such as 6× histidine and Glutathione S-transferase (GST) is frequently used to aid in protein purification [59]. As NMR studies normally require milligrams of protein samples, effort has to be made in the sample preparation step.

#### 2.1.2. Protein Stability

**Figure 2:**
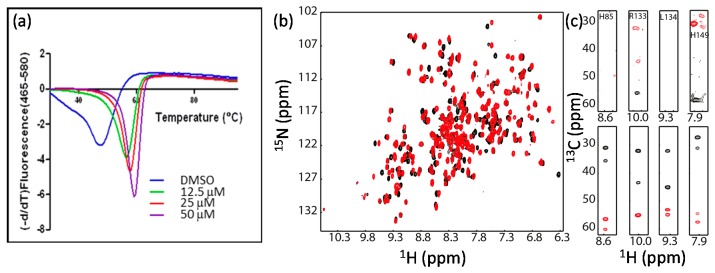
An inhibitor can improve protein stability and be helpful for resonance assignment. (**a**) Inhibitor binding improves protein thermal stability; (**b**) Inhibitor binding induces chemical shift perturbation; (**c**) Inhibitor binding improve the signals of Cα and Cβ. Some selected strips of HNCACB spectra of GyrB in the absence (upper panel) or in the presence (lower panel) of the inhibitor were shown. This figure is obtained from the reference [73] with permission.

Multidimensional heteronuclear NMR experiments are required for resonance assignment. Data collection normally takes from minutes to days, which is dependent on the experimental types and sample concentrations [60]. Although different data collection strategies can be used to reduce data acquisition time [61,62,63,64,65,66], the target protein still needs to be stable during the whole data acquisition period [67]. Buffer conditions such as pH, ion concentration, and other additives such as metal ions can affect protein stability [68,69]. To improve protein stability, the following strategies are normally used. First, the buffer conditions need to be optimized. For example, different proteins may prefer to different pHs. Second, low temperature is preferred for data acquisition as proteins are more stable at lower temperatures, but data collection at a higher temperature can give better sensitivity. Effect of temperature on the protein stability needs to be explored. Third, it is worth the time to make a suitable construct. Various constructs may have effects on protein yield, stability, and spectral quality (see below example) [70,71]. Lastly, whether a known ligand can improve the stability of the target should be evaluated. If a known inhibitor or ligand is available, it normally can improve the protein stability and spectral quality [72]. In the study of N-terminal domain of gyrase B subunit (GyrB) from *Pseudomonas aeruginosa*, it was shown that the free protein was not stable and some residues exhibited weak signals in the 3D-HNCACB spectrum, making the resonance assignment challenging [73]. When an inhibitor was mixed with GyrB, the stability of the complex was increased and the corresponding signals in the spectrum were improved (Figure 2). Resonance assignment for the GyrB complex was obtained, which was used for guiding the assignment of free protein [73]. Therefore, it is also useful for NMR studies when a reference compound is available.

## 3. NMR Experiments Used for Protein-Ligand Interactions

The available NMR experiments that can be used for probing protein-ligand interactions have been described extensively in several reviews [17,37,74,75,76,77,78,79,80] (Table 1). Any experiment that can provide protein and ligand binding information will be useful in drug discovery while choosing a suitable experiment will be dependent on the target size and the experimental purposes. The most commonly used experiment is the chemical shift mapping using a ^15^N-labeled sample because the amide and amide protons are very sensitive to the chemical environments and the required sample is readily to prepare. This type of experiment is suitable for inhibitors with different molecular weight and binding affinities. In this review, we focus on the chemical shift mapping experiment-a widely used experiment to map the ligand binding site and ^19^F-NMR spectroscopy-an efficient tool to identify hits from compound libraries and to prove conformational changes in a target protein.

### 3.1. Chemical Shift Mapping Experiments

The chemical environment of a residue can be affected upon ligand binding, which can cause changes in the NMR spectra. When a protein is isotopically labeled with ^13^C or ^15^N, the chemical environmental changes of residues can be monitored by NMR experiments [17]. Ligand binding changes the chemical environment near the binding site, which will induce the chemical shift perturbations (CSP) of affected residues [119]. Such changes can be observed using ^1^H-^15^N/^13^C-heteronuclear single quantum coherence spectroscopy (HSQC) experiments. Protein-ligand binding information such as binding site and binding mode can be obtained using this type of experiment [81]. Although monitoring chemical shift changes of carbons (Cα, Cβ, C’ and methyl carbons) can be achieved for a protein, monitoring ^15^N chemical shift changes is more frequently used. To determine which residues are affected by ligand binding, resonance assignment is required. Although the backbone assignment can be obtained for proteins with sizes up to over 100 kDa [17], this step is still limited by many factors such as spectral quality and protein stability. Only proteins exhibited well dispersed cross peaks in the spectrum are suitable for further studies. In most cases, for proteins with molecular weight size less than 30 kDa, the assignment can be achieved using conventional 3D-experiments [60]. For large-size proteins with severe signal overlaps in the spectra, other strategies such as selective-isotope-labeling are required for the sequence specific assignment [8,9,120,121,122,123,124].

**Figure 3:**
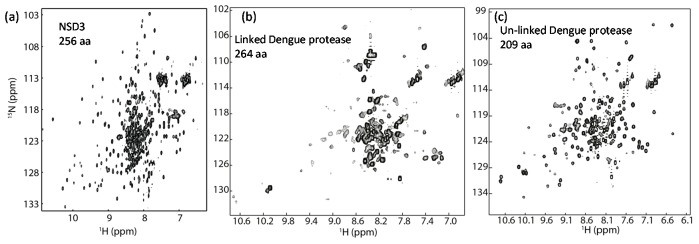
^1^H-^15^N-HSQC spectra of different proteins. (**a**) ^1^H-^15^N-TROSY spectrum of the SET domain of human NSD3; (**b**) ^1^H-^15^N-HSQC spectrum of Dengue virus protease. This is a linked protease construct which may contain both open and closed conformations. The construct exhibits crowded cross peaks in the spectrum; (**c**) ^1^H-^15^N-HSQC of unlinked Dengue virus protease. Removal of the artificial linker in the construct used in (**b**) can result in a protease complex with dispersed cross peaks in the spectrum. The figures are obtained from reference [125] with slight modifications.

Obtaining a good spectrum with dispersed cross peaks is crucial for these types of experiments. Normally protein size affects the quality of the spectrum. Several proteins with molecular weights over 100 kDa have been characterized using NMR spectroscopy. To increase signal sensitivity, TROSY-based [126] experiments are often required for achieving assignment for proteins [73,127,128,129] with size less than 30 kDa. In the backbone resonance assignment of the SET domain of NSD3, ^13^C, ^15^N and ^2^H-labeled sample and TROSY-based experiments [126,130] are essential to obtain the backbone assignment while this construct contains 256 residues (Figure 3a) [131]. Protein dynamics or conformation exchanges may also affect the spectral quality. Presence of dynamic loops may also affect spectral quality. Sometimes it is worth the time to spend some effort to optimize the protein constructs for NMR studies. In the structural study of Bcl-xL antiapoptotic protein by NMR, the length of the loop was reduced, which gives rise to a construct with better stability and higher quality NMR spectra [132]. Such examples also include proteases. For example, the conventional dengue virus protease construct used for structural studies consists of the cofactor region of NS2B and the N-terminal region of NS3 linked via a glycine-rich linker. The linked construct consists of more than 260 residues and exhibits a ^1^H-^15^N-HSQC spectrum with crowded peaks (Figure 3b), which might be due to the existence of open and closed conformations [133]. It is challenging to use such a construct to map the inhibitor binding site by observing CSP because of the conformational exchanges [133]. When a new construct without the artificial linker is made using a co-expression system, the resulting protease construct exhibits resolved cross peaks in the ^1^H-^15^N-HSQC spectrum. Such a construct is more suitable for mapping the inhibitor binding site (Figure 3c) [125]. Therefore, some exploratory studies on optimizing the constructs that can be used for NMR studies should be carried out when the drug discovery project starts.

#### 3.1.1. Differential Chemical Shift

**Figure 4:**
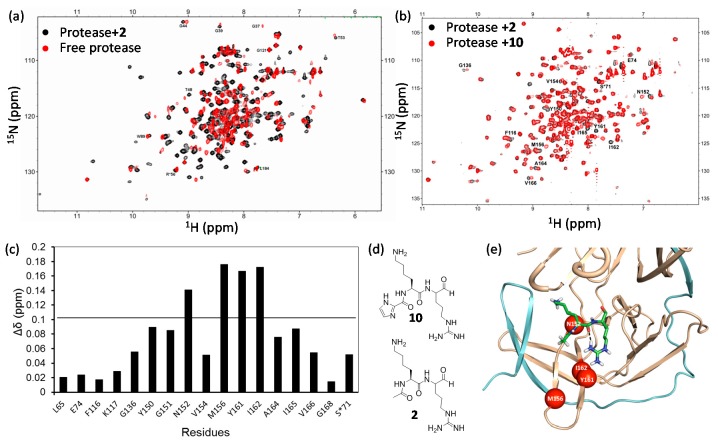
West Nile virus protease and inhibitor interactions. (**a**) Superimposed ^1^H-^15^N-HSQC spectra of protease in the absence (red) and presence (black) of compound **2**; (**b**) Superimposed ^1^H-^15^N-HSQC spectra of protease in the presence of compound **2** (black) and compound **10** (red); (**c**) Chemical shift difference of protease in the presence of **2** and **10**; (**d**) Structures of compound **2** and compound **10**; (**e**) Structural model of West Nile virus protease in complex with **2**. The inhibitor is shown in sticks. The residues exhibit obvious different chemical shifts are shown in red spheres. NS2B and NS3 poly-peptides are shown in cyan and orange, respectively. This figure is obtained from the reference [136] with slight modifications.

CSP provides useful information to identify the ligand binding site, but this method does not provide the orientation information of the ligand [79]. The information is sometimes challenging to be interpreted because the observed changes in chemical shift might also be driven by ligand-induced conformational changes [79]. Of course, obtaining the structure of the complex gives accurate binding information, but structure determination of a complex by NMR is time-consuming and sometimes may not be achievable. It might also be difficult to obtain the crystal structure of the complex. Comparing the chemical shift changes induced by different ligands is a useful way to locate inhibitor binding site and obtain the orientation information of the ligand in its binding pocket. It is not uncommon that quite a few compounds with similar structures are synthesized in the hit-to-lead and lead optimization steps. Therefore, comparing chemical shift changes of a protein induced by several related ligands makes it possible to identify the critical residues for ligand binding and determine the orientation of the ligand in the binding pocket [79]. Successful example was seen in the study of FKBP binding to its ligands. Using this method, the inhibitor binding site on FKBP was unambiguously identified [79]. This strategy was also shown to be very useful for probing the interactions between the West Nile virus protease and the peptidic inhibitors. West Nile virus (WNV) protease is a validated target for developing antivirals. The recombinant protease exhibits a ^1^H-^15^N-HSQC spectrum with well dispersed cross peaks, but not all the residues exhibit detectable peaks due to the conformational exchanges [134]. Protease binding to inhibitor causes significant changes for most of the cross peaks in the ^1^H-^15^N-HSQC spectrum, making it difficult to identify the binding site (Figure 4a). In addition, the chemical shift changes induced by inhibitor binding are difficult to be interpreted because ligand can also stabilize the closed conformation of the protease, giving rise to appearance of new cross peaks in the spectrum [135]. For example, obvious CSPs were observed for most residues when compound **2** was present (Figure 4a). Similar result was observed when protease bound to compound **10**. Almost complete assignment was achieved for the protease-compound **2** complex, but it is difficult to map the binding site as most residues are affected upon ligand binding. To understand the structure activity relationship (SAR) of the inhibitors and locate the inhibitor binding site, the ^1^H-^15^N-HSQC spectra of WNV protease in complexes with several inhibitors with similar structures were compared. Overlapping the ^1^H-^15^N-HSQC spectra of protease in the presence of compounds **2** and **10** demonstrates that only few residues exhibited different chemical shifts (Figure 4b–e). In this case, the inhibitor binding site and the orientation of the inhibitor in the binding pocket can be unambiguously identified. The difference between these two compounds is that compound **10** contains an imidazole moiety. The residues exhibited different chemical shifts should be induced by their interactions with the imidazole in compound **10** (Figure 4c) [136]. Using this approach, the model of the protease and inhibitor complex can be proposed. Although no structure of WNV protease-compound **2** is available, the model was proven by our later structural studies on Zika protease [137]. In addition, this method is very useful for some proteins exhibiting poor spectra in the absence of a ligand. The ligand binding information can be obtained by comparing the spectra of several complexes without referring to that of the free protein. When possible, this method should be applied in drug discovery.

#### 3.1.2. Determining the Binding Affinity

**Figure 5:**
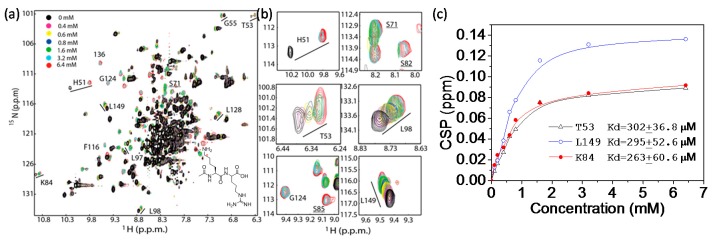
Kd determination using 2D-HSQC-type of experiment. (**a**) ^1^H-^15^N-HSQC spectra of Zika virus protease in the absence and presence of different amounts of dipeptide. Inset is the structure of the peptide used in the experiments; (**b**) Chemical shift changes of several residues. The binding is a complicated process. H51 is undergoing slow or intermediate exchange. Some other residues are undergoing intermediate exchanges and their resonances appeared in the presence of the peptide. Peptide concentration dependent CSP are also observed for several residues close to the protease active site; (**c**) The CSP caused by peptide binding is plotted against peptide concentration. The Kd value for several residues (T53, L149, K84) which are undergoing fast exchange are determined. This figure is obtained from the reference [141] with slight modifications.

In addition to confirm protein-ligand interactions, 2D-HSQC experiment can also be used to characterize the strength of the ligand binding. The position and intensity of the cross peaks behave differently when the ligand binding is in different time scales. If the ligand binds to the target protein strongly, the binding is undergoing slow exchange. The peak intensity of the free protein reduces; the peak of the complex state appears at a different position and the peak intensity will increase when more ligand is added (Figure 5a, H51). If the ligand binds to the target protein weakly, the binding is undergoing fast exchange. As the ligand dissociates from the protein quickly, only the averaged peaks of the free and the bound forms can be observed (Figure 5a, K84 and L149). The peak position will change gradually when the ligand concentration increases. If the binding is undergoing intermediate exchange, both the peak intensity and the peak position would change accordingly.

For binding undergoing slow and intermediate exchanges, it is challenging to obtain the dissociation constant (Kd) value, but the values can be obtained by line shape analysis [138]. The Kd can be calculated based on a series of titration experiments in which an unlabeled ligand is titrated to a labeled protein [139]. This method has been widely used for probing protein-protein, protein-peptide, and protein-ligand interactions which are undergoing fast exchanges. In the titration experiment, additional binding sites can also be identified [140]. To obtain a more accurate Kd value, several residues will be analyzed. Given the fact that the chemical shift of a nucleus such as ^15^N can be affected by many factors such as protein conformations, the Kd values determined using 2D-type experiments may be slightly different from those obtained from other biophysical assays. Nonetheless, such a method is still useful for ranking the binding affinities of different ligands. For example, a dipeptide without the aldehyde group exhibits no inhibitory activity on Zika virus protease in the appropriate biochemical assay. Thermal shift assays reveal that such peptide does not change the thermal stability of the protease [141]. The Kd was then demonstrated to be in mM to μM range using NMR spectroscopy (Figure 5). Although the molecular interactions between protease and the peptide involve multiple events such as conformational changes, the available binding information provided by NMR spectroscopy is helpful for understanding the roles of different functional groups of an inhibitor in protease binding.

Although 2D type experiments can provide amino acid specific binding information, it is a time-consuming procedure and a large amount of labeled protein samples are required. These experiments are more suitable for low-throughput tasks such as fragment screening and hit confirmation. It has been noted that samples can be reused for this type of experiments when the test ligands bind weakly to the target protein. This approach is not practical for high-throughput screening of large compound libraries. In addition to confirming the binding, locating binding site, and determining binding affinity, 2D type experiments are very useful for identifying inhibitors that can break protein-protein interactions by observing the signals from a ^15^N-labeled protein [142].

Ligand-observed NMR experiments have many advantages over the protein-observed experiments [143]. As this type of experiments observe signals from ligands, no isotope-labeling is required for the target protein. These experiments require less acquisition time and can also be used to determine dissociation constants using either titration experiments or by observing changes of the line width of a ligand induced by protein binding. Ligand-observed experiments are very useful for identifying weak-affinity ligand with Kd in the range of μM to mM quantities. For example, Saturation Transfer Difference (STD) [82,83,84], WaterLOGSY experiments [85,86] and Carr-Purcell-Meiboom-Gill (CPMG) sequences [144,145] have been widely used and are well documented in screening. There is no limitation for the size of the target protein. However, ligand-observed experiments are prone to produce false positive results due to nonspecific interaction and aggregation effects [18]. By observing line width changes of a ligand in the absence and presence of a target protein, the binding affinity can be estimated. This approach has been shown to be suitable for HTS [115]. Several reviews and research articles have described the applications of these types of experiments [26,144,145].

### 3.2. ^19^F-Based NMR Experiment

^19^F-NMR experiments for fluorinated organic compounds were carried out in early days [146]. This type of experiment was then used on proteins [147]. ^19^F-based NMR experiments have been widely used in observing protein-ligand interactions, protein conformational changes, or membrane topology of a membrane protein [105,148,149,150,151]. ^19^F-NMR is an attractive approach for probing protein-ligand interactions in drug discovery such as fragment screening because the ^19^F nucleus has a natural abundance of 100% (83% of the sensitivity of ^1^H) and large chemical shift dispersion [148]. The ^19^F atom is not present in biological systems, indicating that no background signal will be present in an assay system [105,151]. To conduct a protein-observed ^19^F-NMR experiment, a target protein should be labeled with a ^19^F atom, which can be achieved using different approaches [152,153]. Several fluorinated amino acids such as the aromatic amino acids 3-fluorotyrosine (3FY), 4-fluoro-phenylalanine (4FF) and building blocks such as 5-fluoroindole are commercially available, and the strategies to incorporate ^19^F into a protein have been described [106,154]. A target protein can be readily ^19^F-labeled in bacterial systems by adding ^19^F-labeled amino acids or precursors in the culture medium [155]. ^19^F atoms can also be incorporated into a target protein by chemical conjugation of fluorine-containing small molecules with residues containing reactive groups such as –SH and NH groups [148]. This can be achieved using the following way. A target protein is first purified. Then ^19^F-containing chemicals such as 2-bromo-*N*-(4-(trifluoromethyl)phenyl)acetamide (BTFMA) can be used to modify the target protein at cysteine residues, resulting in a protein with active ^19^F spins [105,150,151]. For a ligand-observed ^19^F spectroscopy, at least one ^19^F atom should be present in the ligand, which can be easily achieved via chemical synthesis [154].

#### 3.2.1. Hit Identification

^19^F-NMR can be used for hit identification in different ways [156]. First, it can be used in FBDD in which ^19^F-labeled compound libraries are screened using ligand-observed experiments. Several ^19^F-labeled compound libraries for FBDD are even commercially available. These libraries are prepared using similar rules to those used in the normal fragment libraries to sustain ligand size and chemical diversity. The positive hits can be used for further development. Second, ^19^F-NMR can be used for confirming hits screened from HTS campaigns in which a biochemical assay is used as the primary screen [106]. As not all the compounds in the normal HTS library contain ^19^F atoms, the target protein should be labeled with at least one ^19^F atom. Normally, the specific labeled residues should be close to the active site, which can be achieved by referring to biochemical and structural studies. Last, ^19^F-NMR can be used for hit identification/confirmation when a fluorinated substrate is available. This screening assay is more like a competition assay, which requires a careful design. The changes of substrate upon catalysis by the target protein must be monitored by ^19^F-NMR spectroscopy, which can be used to test the effect of screened compounds. Although ligand-observed experiments cannot be used to identify the ligand binding site, protein-observed ^19^F spectroscopy sometimes can be used to identify residues that are critical for binding when the assignments of the ^19^F resonances are available.

#### 3.2.2. Determining Conformational Exchanges

The ^19^F chemical shift is very sensitive to changes in local environment caused by van her Waals interactions and local electrostatic fields [148]. Therefore, ^19^F-NMR can be used to probe protein conformational changes and solvent exposure induced by different types of ligands. In addition to confirming protein-ligand interactions, this method can provide information about conformational exchanges. This method has been shown to be very useful in studies on G protein coupled receptors (GPCRs) which are important drug targets. Based on the available GPCR structures, different types of ligands can cause conformational changes which can be further confirmed by ^19^F-NMR [105,157]. As GPCRs are membrane proteins, expression of the target protein is normally achieved in eukaryotic systems such as yeast, insect cells, or mammalian cells. The target protein was first purified in a membrane system, followed with chemical conjugation of ^19^F-labels [157]. Also assignments of the ^19^F resonances can be assigned by a mutagenesis method when multiple modification sites are available. In the study of β_2_AR, the target protein contains three native cysteine residues (C265, C327, and C341) [151]. Covalent labeling of β_2_AR with trifluoroethylthio (TET) results in a sample exhibiting three peaks in the corresponding 1D ^19^F-NMR spectrum. Sequence-specific assignments of these peaks were then achieved by site-specific mutations. Cysteine residues can also be introduced into certain residues for attaching ^19^F labels. Special care has to be taken to make sure that mutations or modifications still preserve the biological activities of the target protein. Using ^19^F-NMR, the activation of GPCRs upon ligand binding can be evaluated. This method is also very applicable for water soluble proteins. In the study of dengue virus protease, a ligand can induce the closed conformation, which can be easily evaluated by ^19^F-NMR spectroscopy [158]. It has been noted that this method will be every applicable when the structural information of the target protein in the absence and presence of ligands is available.

#### 3.2.3. Ranking Compound Binding Affinities

**Figure 6:**
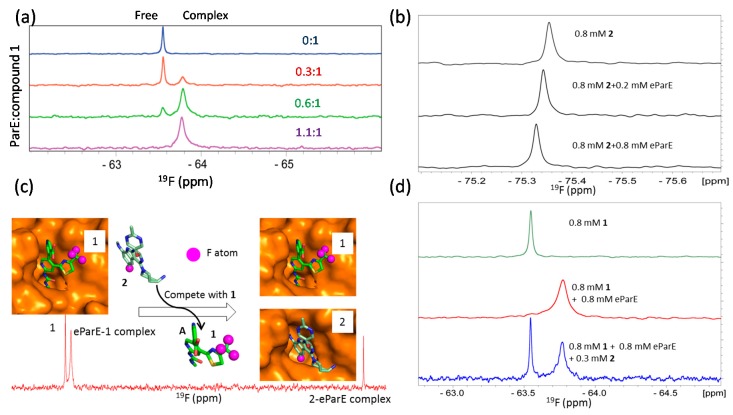
^19^F NMR in competition studies. (**a**) ^19^F NMR of compound **1** in the presence of different amounts of eParE. Signal from free and eParE bound compound **1** can be observed and exhibit obviously different chemical shits; (**a**) is obtained from the reference [161] with slight modifications; (**b**) ^19^F NMR of compound **2** in the presence of different amounts of eParE; Chemical shift perturbation is observed for compound **2** when it binds to eParE; (**c**) Compound **2** can displace compound **1** out of the binding pocket. The models of the eParE in complexes with compounds **1** and **2** are shown, respectively. ^19^F atoms are labeled as purple spheres; (**d**) ^19^F NMR of compound **1** in the equal molar concentration of eParE and 0.3 mM of compound **2**. Compound can be competed from eParE as resonance of free compound **1** is observed when compound **2** is present; (**b**–**d**) is obtained from the reference [163] with slight modifications.

The NMR reporter screening is a novel technique where a reference compound is used to screen or identify ligands with higher binding affinities to the target [114]. This method observes ligand signals which can be affected by the presence of the target. In a normal 1D proton or ^19^F spectrum, signals from the reference compound are affected (line broadening or chemical shift changes) in the presence of the target protein. When the test compounds are added into the mixture, NMR spectra of free reference compound and its complex with the target were acquired and compared. The test compound will then be confirmed to interact with the target by replacing the reference compound from its binding pocket of the target if the NMR signals of the mixture are same as those of the free reference compound [114]. As this method is very useful for screening ligands with higher affinities than that of the reference compound, it is therefore necessary to know the detailed binding information between the target and the reference molecule [114,159]. This competition experiment can also be used for HTS and using a ^19^F-labeled molecule as a reference [78] or using proton-based experiments using a normal ligand [160]. The competition experiment using ^19^F probe can be easily used in hit identification and lead optimization steps as this method ranks the binding affinities of the test compounds. Comparison of binding affinities of a series of compounds generated from a lead can be easily achieved using a normal ^19^F-NMR spectrum. The reference compound has to be selected carefully in this study because the chemical shift changes induced by protein binding may be different for different compounds. For example, both a bis-pyridylurea inhibitor (compound **1**) and an inhibitor (compound **2**) with a 9*H*-pyrimido[4,5-*b*]indole scaffold bind to the active site of *E. coli* topoisomerase IV E subunit (eParE) with binding affinities of 902 nM and 1.14 nM, respectively. Both compounds are potent inhibitors and the bindings undergo slow exchange based on the titration experiments monitored using ^1^H-^15^N-HSQC spectra [73,161,162,163]. Both compounds contain ^19^F groups with different binding profiles upon binding to eParE. Compound **1**, with low eParE binding affinity (902 nM), exhibits two obvious peaks corresponding to free and eParE-bound resonances in its 1D ^19^F spectrum when a small portion of eParE is present (i.e., the protein to ligand ratio less than 1). It is straightforward to monitor the binding event as the resonance corresponding to the protein bound form can be easily identified. On the contrary, compound **2** has a higher binding affinity (1.14 nM) with eParE than compound **1**. Only a moderate CSP was observed in the ^19^F spectrum when it was titrated with eParE (Figure 6). This may be due to the chemical environment of the ^19^F atoms in compound **2**. Compound **1** is then very useful for the competition experiment to identify compounds having higher binding affinities. To conduct such an experiment, compound **1** was first mixed with equal amount of eParE and the resulting mixture generates a ^19^F spectrum with signals from the complex (Figure 6). When compound **2** was added to the mixture, compound **1** is competed out of the binding pocket and the signal of free compound **1** appears in the spectrum (Figure 6). It has been noted that compound **1** is useful in the lead optimization step because of its high binding affinity with eParE. A different reference molecule is needed if ^19^F-NMR is going to be used for fragment-based screening as the binding affinities of the fragments are normally weaker than the lead compounds. Many fragments targeting this class of enzymes are available [164]. Incorporation of an F atom in a compound is not a complicated procedure. Therefore, a reference compound can be easily generated for competition experiment when some known ligands are available.

## 4. Solution NMR in Target Engagement

Target engagement is important in both drug discovery and chemical biology. It is a procedure to probe the binding of a drug candidate or a small molecule to its protein target in a living cell [165]. Target engagement for the developed leads is crucial for proving the clinical hypothesis as it is important to make sure that the developed compounds hit the desired target in the living cells, animal models and patients. Target engagement in living cells during the lead optimization step is preferred as the experiments can be easily carried out in lower cost cell-based assays. Several methods such as cellular thermal shift assay [166,167] and polarized microscopy [168] have been used for target engagement. In-cell NMR spectroscopy allows the structural study of proteins in living cells [169]. In-cell NMR studies were first carried out in *E. coli* in which targets of interest can be overexpressed [169,170]. Protein structures can be determined in living cells using multidimensional experiments [171]. Data acquisition times can also be reduced using a non-uniform sampling data collection scheme [172]. Protein-protein interactions [173] and the behavior of intrinsically disordered proteins [174,175] can be evaluated in cells using this approach. In eukaryotic cells, the first in-cell NMR experiments were carried out on labeled proteins that were injected into the oocytes of *Xenopus laevis* [7,176,177]. Using cell-penetrating peptides, an isotopically labeled protein can be delivered into living human cells. The target proteins can be released from the peptides by endogenous enzymatic activity or by autonomous reductive cleavage [178]. NMR spectra can be collected and protein-ligand interactions can be monitored in the living cells [178]. In recent years, in-cell NMR was utilized for probing protein structures, disulfide-bond formation, and metal uptake in living cells [172,179,180,181]. It was normally very challenging to express isotopically labeled proteins in mammalian cells. A method has been described to overexpress one or several proteins in human embryonic kidney 293T (HEK293T) cells using transient DNA transfection to achieve uniform ^15^N-labeling for heteronuclear experiments [182]. To our knowledge, there is no report regarding target engagement using solution NMR spectroscopy in a drug discovery process. In-cell NMR has been used in probing protein folding and modification [183] in living mammalian cells and screening compound libraries [184,185]. Although it is challenging to probe protein and ligand interactions in living cells due to many difficulties such as the poor spectral quality arising from specific and non-specific interactions, the recent progress has shown the potential application of this technique. When a target protein is able to exhibit detectable and nicely dispersed NMR spectra, in-cell NMR will be a very powerful tool to conduct target engagement.

## 5. Conclusions and Perspectives

NMR is a powerful tool in drug discovery because of its roles in probing protein-ligand interactions in solution. With the availability of newly developed pulse programs and high-field magnets, it is possible to investigate high-molecular weight protein targets. It is time-consuming to determine protein solution structures while most drug discovery projects have a timeline. NMR spectroscopy is therefore very useful for confirming ligand binding, mapping ligand binding interfaces and fragment-based drug discovery using 1D or 2D type experiments. ^19^F-NMR spectroscopy is particularly useful in drug discovery because of its high sensitivity, which requires less data acquisition and processing time. Ligand- and protein-observed ^19^F-NMR spectroscopy should be considered in a drug discovery project if it is possible. In-cell NMR can also play an important role in drug discovery by providing binding information in living cells.

## Figures and Tables

**Table 1:** List of commonly used NMR experiments for probing protein-ligand interactions.

Experiments	Protein Labeling	References
^1^H-^15^N/^13^C-HSQC	^15^N/^13^C	[17,81]
Saturation Transfer Difference	NA ^1^	[82,83,84]
WaterLOGSY	NA	[85,86]
Transferred NOESY Experiment	^13^C, ^15^N, or NA	[87,88,89,90,91,92,93,94]
Filtered NOESY	^13^C, ^15^N	[95,96,97,98,99]
Residue Dipolar Coupling	^15^N, ^13^C, or NA	[100,101,102]
Ligand-observed ^19^F-NMR	NA	[103,104]
Protein-observed ^19^F-NMR	^19^F	[105,106,107]
Cross-saturation	^15^N	[108,109]
Paramagnetic Relaxation Enhancement	^15^N, ^13^C/^15^N	[50,110,111]
H-D exchange	^15^N	[112,113]
NMR reporter screening/competition assay	NA	[114,115]
Relaxation and relaxation dispersion	^15^N	[116,117,118]

^1^ NA means no isotopic labeling is required.

## Abbreviations

The following abbreviations are used in this manuscript:

NMR	Nuclear magnetic resonance
HTS	High-throughput screening
FBDD	Fragment-based drug discovery
CSP	Chemical shift perturbation
HSQC	Heteronuclear single quantum coherence spectroscopy
*E. coli*	*Escherichia coli*
GST	Glutathione S-transferase
GyrB	gyrase B subunit
eParE	*E. coli* topoisomerase IV E subunit
GPCR	G protein coupled receptor
WNV	West Nile Virus
